# Effects of dose de‐escalation following testosterone treatment and evoked resistance exercise on body composition, metabolic profile, and neuromuscular parameters in persons with spinal cord injury

**DOI:** 10.14814/phy2.15089

**Published:** 2021-10-29

**Authors:** Ashraf S. Gorgey, Refka E. Khalil, Ranjodh Gill, Rehan Khan, Robert A. Adler

**Affiliations:** ^1^ Spinal Cord Injury and Disorders Center Hunter Holmes McGuire VAMC Richmond Virginia USA; ^2^ Department of Physical Medicine & Rehabilitation Virginia Commonwealth University Richmond Virginia USA; ^3^ Endocrinology Service Hunter Holmes McGuire VA Medical Center Richmond Virginia USA; ^4^ Endocrine Division Virginia Commonwealth University School of Medicine Richmond Virginia USA; ^5^ Radiology Service Hunter Holmes McGuire VA Medical Center Richmond Virginia USA

**Keywords:** basal metabolic rate, body composition, dose de‐escalation NMES, glucose effectiveness, inflammatory and anabolic biomarkers, resistance training, spinal cord injury, testosterone treatment, visceral adipose tissue

## Abstract

The dose de‐escalation (DD) effects of testosterone and evoked resistance training (RT) on body composition, cardiometabolic, and neuromuscular variables were investigated. Thirteen men with chronic complete spinal cord injury (SCI) were followed for additional 16 weeks after receiving either testosterone treatment only (TT) or TT+RT. During the 16‐week DD period, the TT+RT group underwent a program of once weekly electrical stimulation with gradually decreasing ankle weights and testosterone patches of 2 mg day^−1^ (TT+RT group). The TT only group did not receive any intervention throughout the detraining period (no‐TT group). Body composition was tested using anthropometrics, dual energy X‐ray absorptiometry, and magnetic resonance imaging. After an overnight fast, basal metabolic rate (BMR), lipid panel, serum testosterone, inflammatory biomarkers, glucose effectiveness, and insulin sensitivity were measured. Finally, peak isometric and isokinetic torques were measured only in the TT+RT group. All measurements were conducted at the beginning and at the end of DD. Absolute thigh muscle cross‐sectional areas (CSAs) demonstrated interaction effects (*p* < 0.05) between the TT+RT (−8.15%, −6.5%) and no‐TT (2.3%, 4.4%) groups. Similarly, absolute knee extensor muscle CSA demonstrated interaction effects (*p* < 0.05) between the TT+RT (−11%, −7.0%) and no‐TT (2.6%, 3.8%) groups. There was a trend (*p* = 0.07) of increasing visceral adipose tissue (VAT) CSAs in the TT+RT (18%) and in the no‐TT (16% cm^2^) groups. There was an interaction (*p* = 0.005) between TT+RT (decreased by 3.7%) and no‐TT groups (increased by 9.0%) in BMR. No interactions were evident between groups over time for biomarkers related to carbohydrate, lipid metabolism, or inflammation. Finally, there were no changes (*p* > 0.05) in peak isometric or isokinetic torques and rise time following 16 weeks of the DD period in the TT+RT group. TT+RT during 16 weeks of DD was minimally effective at preventing detraining relative to no‐TT on muscle size, BMR, and VAT. However, neuromuscular gains were successfully maintained.

## Graphical abstract

Sixteen weeks of dose de‐escalation program did not adequately maintain the gain in muscle size and basal metabolic rate. However, it prevents detrimental changes in neuromuscular adaptations.

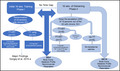

## 1 INTRODUCTION

Surface neuromuscular electrical stimulation (NMES) evokes skeletal muscle hypertrophy, restores lean mass, decreases percentage fat mass, and enhances cardiometabolic profile in persons with spinal cord injury (SCI; Dudley et al., 1999; Gorgey, Khalil, et al., 2019; Mahoney et al., 2005). NMES has been shown to elicit significant improvements in whole‐body cardiometabolic profiles (Gorgey, Khalil, et al., 2019). Several studies demonstrated that training the large paralyzed lower extremity muscles is likely to be associated with improvements in peak oxygen uptake, lipid, and carbohydrate profiles as well as bone mineral density in persons with SCI (Dolbow et al., 2011). To maximize the benefits on cardiometabolic risk factors, NMES‐resistance training (NMES‐RT) was successfully combined with testosterone in eugonadal men with complete SCI (Gorgey, Khalil, et al., 2019). Combining NMES‐RT with testosterone resulted in increasing whole thigh and knee extensor muscle cross‐sectional area (CSA) by 29.5% and 43%, respectively, without changes in the testosterone only (testosterone treatment [TT]) group (Gorgey, Khalil, et al., 2019). Both groups showed modest decreases in visceral adipose tissue (VAT) and interleukin 6 (IL‐6) with a decrease in intramuscular fat (IMF) in the TT+RT group. Basal metabolic rate (BMR) increased by 211–250 kcal/day only in the TT+RT group (Gorgey, Khalil, et al., 2019). These findings supported earlier work which demonstrated that 5–10 mg/day of TT for 12 months increased serum testosterone level from 251 to 504 ng/dl, increased lean mass and resting metabolic rate in hypogonadal men with SCI (Bauman et al., 2011).

Limited evidence in persons with SCI exists regarding how training cessation affects cardiometabolic risk factors and neuromuscular parameters such as muscle peak torque, rise time, and fatigue (Bauman et al., 2015; Gorgey, Martin, et al., 2016; Holman & Gorgey, 2019). In able‐bodied controls, the effects of 4‐week detraining on neuromuscular parameters were tested following 8 weeks of NMES training (Gondin et al., 2006). The authors noted decreases in knee extensor maximum voluntary contraction, vastii muscle electromyography activity, muscle activation, and quadriceps CSA by 9%, 20%, 5%, and 3%, respectively (Gondin et al., 2006). Bickel et al. reported that exercise dose de‐escalation (DD) was capable of maintaining certain RT adaptations during a 32‐week detraining period (Bickel et al., 2011). The DD RT program was accomplished by reducing the training volume to 1/3 (three sets of 10 once a week) and 1/9 (one set of 10 once a week) from the original three sets thrice weekly (Bickel et al., 2011). Interestingly, older individuals (60–75 years) required a higher maintenance dose to retain the gain in myofiber CSA and myofiber type transformation (Bickel et al., 2011).

In persons with chronic SCI, 2.5 years after cessation of either arm cycling exercise or functional electrical stimulation cycling leg lean mass and whole‐body lean mass decreased by 16% and 5.4%, respectively, with a 15.5% concomitant decrease in BMR following (Gorgey, Martin, et al., 2016). Similarly, Gurney et al. showed that 8 weeks of detraining following 12 weeks of functional electrical stimulation cycling resulted in a 22.5% decrease in peak oxygen uptake and a 52.5% decrease in peak workload (Gurney et al., 1998). In contrast, Bauman et al. showed that lean tissue mass and energy expenditure were retained for an additional 6 months in hypogonadal men with SCI following discontinuation of 12 months of transdermal testosterone application (Bauman et al., 2015). The authors suggested persistent beneficial effects of anabolic hormone therapy on lean mass and resting metabolic rate in men with SCI (Bauman et al., 2015). Therefore, the addition of testosterone to NMES‐RT may result in retention of the cardiometabolic benefits or neuromuscular parameters after cessation or DD period in persons with SCI.

Previous studies primarily relied on administering a period of passive detraining (i.e., no intervention following an active exercise program). A limited number of studies have focused on investigating the effects of DD (i.e., reducing the frequency or volume of training, or the dose of administered medication). Reducing the frequency of training has previously been recommended as an effective strategy to enhance adherence and compliance to a longitudinal exercise programs (Gorgey et al., 2016). In our previous attempts, we have utilized a frequency of twice weekly of NMES‐RT to restore muscle mass and enhance the cardiometabolic profile (Gorgey, Khalil, et al., 2019) and neuromuscular parameters (Holman & Gorgey, 2019). Even a frequency as low as once weekly of NMES‐RT may result in increased leg lean mass and reduced muscle fatigue in a person with T6 SCI (Gorgey, Caudill, et al., 2016). Therefore, a DD period of once weekly NMES‐RT combined with testosterone might retain the cardiometabolic benefits and neuromuscular parameters after 16 weeks of training.

The primary purpose of the current study was to investigate the effects of 16 weeks of DD with low‐dose testosterone and NMES‐RT (TT+RT) on parameters of body composition, cardiometabolic profiles, and neuromuscular parameters compared to no‐TT (i.e., 16 weeks without any testosterone or NMES‐RT) in men with chronic complete SCI. In the current study, the TT+RT group underwent a decrease in the dose of testosterone and the volume of NMES‐RT during the DD period. For the TT+RT, the DD program was designed by reducing the training volume (four sets of 10 to three sets of 10) and the training frequency (twice weekly to once weekly) from the original 16‐week training (Gorgey, Khalil, et al., 2019) and similar to earlier recommendations intended to maintain training adaptations (Bickel et al., 2011). Additionally, the DD program introduced the lowest dose of testosterone (2 mg day^−1^) in the TT+RT group and ceased administration of testosterone in the TT group (no‐TT) similar to earlier work (Bauman et al.2015). Our primary hypothesis was that decreasing the dose of testosterone combined with NMES‐RT in the TT+RT group may mitigate the effects of detraining on muscle size, VAT, neuromuscular parameters (peak torque and rise time), and cardiometabolic profiles compared to cessation of testosterone in the no‐TT group after chronic SCI.

## 2 METHODS

**FIGURE 1:**
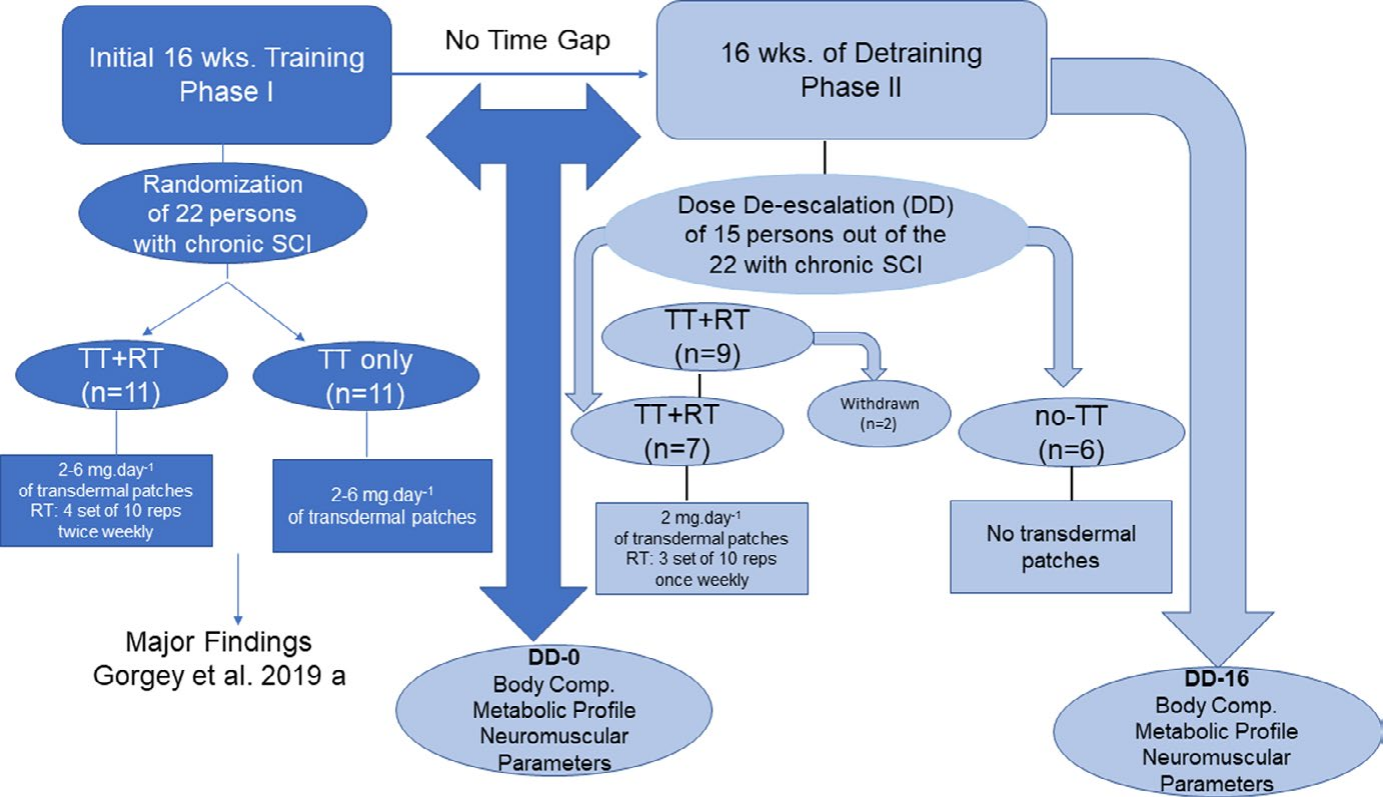
Timeline of phase I (effects of TT+RT vs. TT only) and phase II (DD of TT+RT vs. no‐TT) on cardiometabolic risk factors and neuromuscular parameters in persons with chronic SCI. Dark blue reflects intervention in phase I (Gorgey, Khalil, et al., 2019) and light blue reflects the DD phase. DD, dose de‐escalation; RT, resistance training; TT, testosterone treatment

Fifteen men with complete SCI, who were originally randomized into 16‐week open‐label manner to investigate the effects of TT+RT compared to TT only on body composition and metabolic profile (Gorgey, Khalil, et al., 2019) and neuromuscular parameters (Holman & Gorgey, 2019), were invited to participate in an additional 16 weeks of DD period. Originally, both groups received 16 weeks of transdermal testosterone patches (2–6 mg day^−1^) that were alternated between left and right shoulders at bedtime. Additionally, the TT+RT group received 16 weeks of supervised progressive RT, twice weekly using surface NMES and ankle weights (Gorgey, Khalil, et al., 2019). After maintaining each group's original assignment, only 13 men were followed for 16 weeks to investigate the effects of DD of TT+RT (*n* = 7) and no‐TT (*n* = 6) on cardiometabolic risk factors and neuromuscular parameters. Two participants withdrew from the TT+RT group (see the Section 3). There was no time gap between the initial phase of the trial (Gorgey, Khalil, et al., 2019) and the second phase of DD. This additional 16‐week period started (DD0) and concluded (DD16) with a 2‐day visit consisting of an overnight stay for measurements of body composition, metabolic profile, peak isometric, and isokinetic torques. DD0 corresponds to the post‐intervention one time point in the original trial (Gorgey, Khalil, et al., 2019). The design of the training and DD program is illustrated in Figure 1.

### 2.1 Consenting and physical examination

The study was approved by the local institutional research board. After signing an informed consent, each participant underwent a detailed physical examination by a trained physician. Detailed inclusion and exclusion criteria as well as the process of recruitment and randomization were previously described (Gorgey, Khalil, et al., 2019). Briefly, participants underwent physical examination that included a neurological assessment, electrocardiogram, and International Standards for Neurological Classification of Spinal Cord Injury.

### 2.2 Interventions

#### 2.2.1 Resistance training using evoked NMES

The RT protocol using surface NMES was recently described in detail and shown in a video publication demonstrating step‐by‐step strategies to effectively implement surface NMES in persons with SCI (Mahoney et al., 2005; Ryan et al., 2013). Briefly, one surface adhesive electrode was placed on the knee extensor muscles 2–3 cm above the superior aspect of the patella over the vastus medialis muscle, and the other adhesive electrode was placed lateral to and 30 cm above the patella over the vastus lateralis muscle. A Theratouch 4.7 stimulator unit (Rich‐Mar**)**, was set to deliver biphasic rectangular pulses of 30 Hz, 450 µs pulses at a current amplitude (mA) sufficient to evoke full leg extension against gravity. The current was manually increased to evoke full leg extension (three sets of 10 repetitions) with a 2–3‐min rest between sets as previously described (Gorgey, Khalil, et al., 2019). The sets were alternated between the right and left leg starting with the right leg and the current amplitude (mA) was recoded for every repetition. Training was conducted once weekly for 16 weeks with participants sitting in their own wheelchairs. Training started with the maximum ankle weights that were achieved during the original 16‐week trial and was gradually decreased by 2 lbs. (0.91 kg) per week until ankle weights reached 2 lbs. and were maintained for the remaining weeks. Training ensured full knee extension was achieved for 30 reps without fatigue.

#### 2.2.2 Testosterone treatment

The TT+RT group applied 2 mg day^−1^ testosterone shoulder patches (Androderm, Watson Pharma. Inc.; Gorgey, Khalil, et al., 2019) while the TT only group did not apply testosterone patches during the DD period (no‐TT group). The 2 mg day^−1^ was chosen because it is the minimal dose according to the manufacturer. Patches were supplied every 30 days and returned on a monthly basis to ensure adherence to the intervention protocol. Participants were instructed to place patches before bedtime and keep it on for 24 h. Patches were removed in the morning before bathing and re‐attached on the same spot for the rest of the day. Participants discontinued T patches on the last day of the study or 4 days prior to DD16 measurements.

##### Two‐day assessment period

The 2‐day assessment period included measurements of body composition, anthropometry, dual energy X‐ray absorptiometry (DXA) as well as measuring peak isometric and isokinetic torques. Additionally, magnetic resonance imaging (MRI) scans were obtained for whole thigh, individual skeletal muscles and IMF CSAs, trunk VAT, and subcutaneous adipose tissue (SAT). On the day of body composition assessment, participants were reminded to consume adequate fluids to stay hydrated and to eat a light meal 2–3 h prior to testing (Dixon et al., 2013). Participants were then escorted to the clinical research unit for the dinner and remained in the unit overnight for metabolic measurements the following morning.

###### Anthropometrics and body composition assessments

The height of each participant was determined while lying in a supine position using their left sides (Wade & Gorgey, 2017). Two smooth wooden boards were placed at the participant's head and heels and the distance between them was measured to the nearest cm. Measurements of abdominal girth (widest region of the trunk), waist (narrowest region of the trunk), hip (encompasses both greater trochanters), and thigh (mid‐point between anterior superior iliac spine and superior border of the patella) circumferences were measured in triplicate in the supine position (Wade & Gorgey, 2017). For the first three circumferences, participants were asked to take a deep breath and then exhale, and measurements were captured at the end of expiration. Measurements were repeated if there was a difference greater than 0.5 cm between repeated readings (Gorgey, Martin, et al., 2016).

###### Dual energy X‐ray absorptiometry

Body composition was measured by whole‐body scans using a GE Lunar Prodigy Advance scanner (GE Lunar Inc.). Fat‐free mass (FFM), fat mass (FM), %FM, and LM for total body, trunk, legs, arms, android, and gynoid regions were measured by DXA (Gorgey et al., 2018; Spungen et al., 2003). The DXA scanner was calibrated using a daily quality control phantom according to the manufacturer’s guidelines. Participants were transferred to the DXA table using either a ceiling lift or self‐transfer with or without sliding board. Participants were allowed 20 min in a flat supine position to account for possible fluid shifts before starting the scan. Knees were strapped together using a large velcro strap above the knee joints and every effort was made to ensure that each leg was placed in a neutral position with the big toe facing upward. The lead research investigator checked that the whole‐body posture was aligned straight with no rotation in the pelvis or shifting of the trunk. The arms were placed close to the body in mid‐prone position to ensure the total body was within the scanning field (Gorgey et al., 2018). All scans were performed and analyzed by a trained DXA operator using Lunar software version 10.5. Total regional borders were placed by the computer auto analysis program delineating anatomical regions of interest and final adjustments were made to ensure optimum inter‐participant reproducibility. We have reported the short‐and long‐term precision of the regional and whole‐body composition using DXA in persons with SCI (Gorgey et al., 2018).

###### Magnetic resonance imaging

*Thigh* *muscle CSA (Primary outcome variables)*: Magnetic resonance imaging was performed at the VA Medical Center using a General Electric Signa 1.5‐T magnet as previously described (Elder et al., 2004; Gorgey, Khalil, et al., 2019; Wade & Gorgey, 2017). Transaxial images (12–15 slices; fast spin echo; repetition time, 850–1000 ms; echo time, 6.7 ms; imaging frequency, 63.8 MHz; echo number, 1; echo train length, 3; flip angle, 90°; field of view, 20 cm; matrix size, 256 × 256) 8 mm thick and 16 mm apart, were taken from the hip joint to the knee joint using a General Electric body array flex coil to measure thigh CSA. Using a localized coil, the signal‐to‐noise ratio was improved, resulting in high‐resolution images for analysis. The acquisition time per leg was 3.5 min. The participant's legs were strapped together to mitigate involuntary muscle spasms, and participants were provided earplugs to minimize the noise. Images were analyzed using Win‐Vessel software (Ronald Meyer, Michigan State University). To distinguish muscle from fat, the outer perimeter of the thigh muscle group was manually traced, and pixel signal intensity was automatically determined via the software. A bimodal histogram segmentation was plotted that contained two distinct peaks, with the first peak representing the threshold for muscle and the second peak representing the threshold for fat. This mid‐point value was used to separate muscle pixels from IMF pixels as previously described (Gorgey, Khalil, et al., 2019).

**FIGURE 2:**
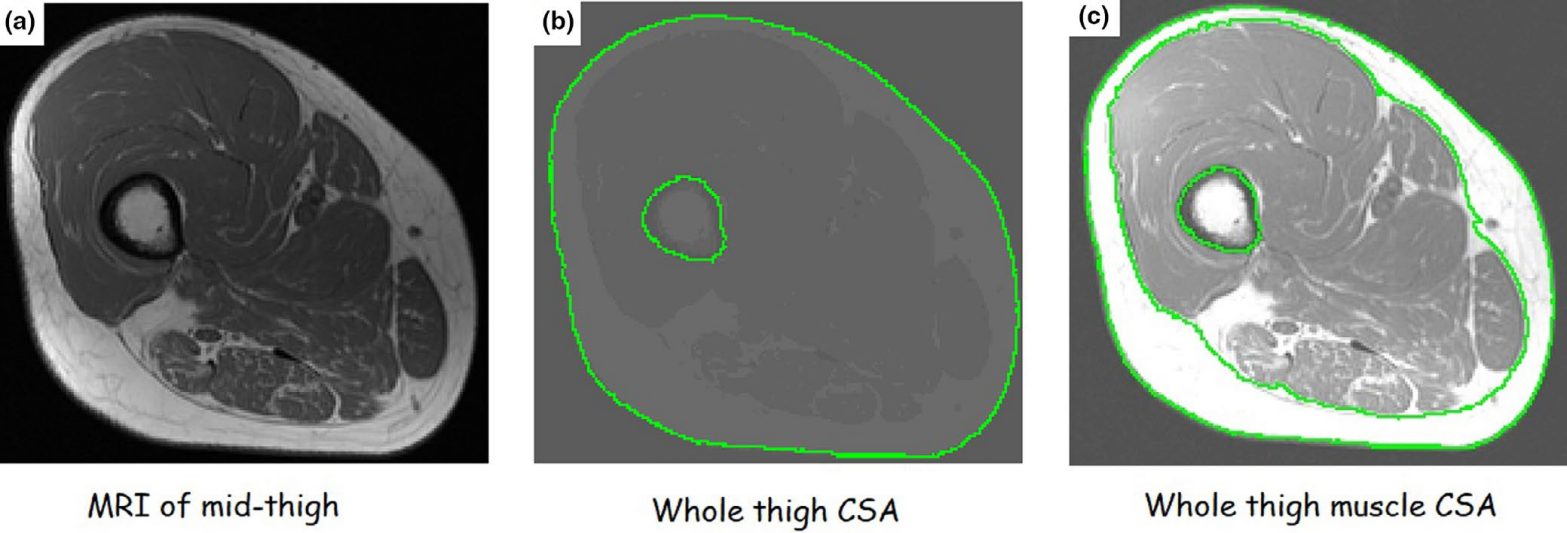
Representative MRI images of the mid‐thigh showing a step‐by‐step procedure of capturing and analysis (a) raw image; (b) whole thigh CSA after segmentation and tracing on the outside subdermal border and excluding the bone CSA. The whole thigh CSA includes thigh subcutaneous adipose tissue (SAT) and whole thigh muscle CSA; (c) whole thigh muscle CSA is measured after tracing on the deep subfascial border after excluding SAT (i.e., white adipose tissue surrounded by the two large green circles) and bone CSAs. The whole thigh muscle CSA includes absolute muscle CSA and intramuscular fat (i.e., white adipose tissue infiltrated within the anatomical boundaries of different muscle groups and inside the inner green circle). CSA, cross‐sectional area; MRI, magnetic resonance imaging

Regions of interest were manually traced including whole thigh CSA (thigh CSA = muscle CSA+SAT CSA; Figure 2a). Whole skeletal muscle CSA is the entire thigh muscle CSA including IMF and excluding bone CSA (Figure 2b). Absolute skeletal muscle was determined via signal intensity after excluding IMF and femoral bone CSA. SAT CSA was defined as the area between the outside of the muscle CSA and inside of the thigh CSA (Figure 2c).

*Visceral* *and subcutaneous adiposity*: Magnetic resonance imaging images were obtained using a Echelon RAPID Torso/Body Coil (Hitachi Medical Systems America) to capture multiaxial slices of the trunk region (Gill et al., 2020; Sumrell et al., 2018). Transverse axial images (axial in‐phase/out‐phase with a repetition time of 140 ms and echo time of 4.2 and 2 ms for the in‐phase and the out‐phase, respectively; a 42‐cm field of view; matrix size of 256 × 256; one number of excitation; acquisition time of 40 s and slice thickness was 0.8 cm and interslice space was 0.4 cm) were obtained from the xiphoid process to L4–L5 and from L4–L5 to the femoral heads. In the supine position, subjects had lower extremities strapped to avoid unpredictable movement due to spasms during the scan and subsequent image artifacts. Participants were instructed to maintain their position during the scan and were asked to take a deep breath and hold it for 20 s, to prevent any respiratory artifacts that could alter the quality of images.

Visceral adipose tissue was measured across different anatomical regions of the trunk between liver and kidneys (VAT_L‐k_), between kidneys and umbilicus (VAT_K‐U_), between iliac crests and femoral heads (VAT_IC‐FH_), and total VAT (VAT_total_; Gorgey, Khalil, et al., 2019). TT patches were removed 48–72 h prior to MRI scans to avoid possible skin burn.

##### Day 2‐Metabolic testing (secondary outcome variables)

After completing the body composition assessment, participants were then escorted to the clinical research unit for dinner and remained there overnight for metabolic studies the following day.

###### Basal metabolic rate

After an overnight fast of 10–12 h, participants were kept in a dark room for 20–30 min to attain a resting state during which BMR was measured as previously described (Nightingale & Gorgey, 2018). Briefly, while in the supine position a canopy was placed over the subject's head. Each subject was allowed 2–3 min before starting the test to ensure that subjects were calm and comfortable prior to initiating measurements. All subjects were instructed to stay awake during the entire test and to breathe normally. The canopy was then attached to a vacuum to draw expired gases to the flowmeter of the metabolic unit (COSMED KB42; COSMED). Prior to the test, the metabolic unit was calibrated using standard procedures as recommended by the manufacturer. Carbon dioxide and oxygen output were used to calculate the respiratory exchange ratio, and the BMR (kcal/day) was calculated using the average of the last 15 min of the test.

###### Serum T, anabolic growth factors, lipid panel, adiponectin, and inflammatory biomarkers

After BMR, fasting blood samples were collected at approximately 6.30 a.m. Total testosterone was measured by liquid chromatography with isotope dilution mass spectrometry detection after supported liquid extraction (ESOTERIX INC.). The amount of testosterone in each sample was calculated from a linear plot generated by purified testosterone standards ranging from 2.5 to 5000 ng/dl.

Fasting lipid panel (high, low densities lipoprotein cholesterols, total cholesterol, and triglycerides) was determined using enzymatic colorimetric assays. Inflammatory biomarkers C‐reactive protein (CRP), IL‐6, Tumor necrosis factor (TNF)‐α, and free fatty acids were determined by commercially available enzyme‐linked immunosorbent assay kits (ALPCO).

###### Intravenous glucose tolerance test

After fasting blood samples, an intravenous line was placed to facilitate infusion of glucose and blood sampling (Gorgey, Khalil, et al., 2019; O'Brien et al., 2017). Blood samples were taken before and every 2–3 min after glucose injection (0.3 gm/kg IV over 30–60 s) for 30 min, followed by blood collection every 5–10 min ending at 180 min after glucose injection. Twenty minutes after the glucose injection a bolus of insulin (0.02 U/kg, regular short acting insulin, Humulin; Lilly) was injected to determine insulin sensitivity. Plasma glucose was measured by the autoanalyzer glucose oxidase method and plasma insulin concentrations were determined by commercial radioimmunoassay (ALPCO). The glucose disposal rate per unit of secreted insulin per unit time and glucose‐mediated glucose disposal rate were calculated from a least squares fitting of the temporal pattern of glucose and insulin throughout the intravenous glucose tolerance test (IVGTT) using the MINMOD program. The insulin sensitivity index (Si) describes the effect of insulin to promote glucose disposal and to inhibit hepatic glucose production. Glucose effectiveness (Sg) indicates the ability of glucose to cause its own uptake into the cell at basal insulin levels.

##### Peak isometric and isokinetic torques (TT+RT only)

Briefly, peak isometric and isokinetic peak torques were measured using a Biodex Isokinetic Dynamometer after transfer with an Arjo barrier‐free from the wheelchair to the Biodex System (Holman & Gorgey, 2019) only for the TT+RT group. We could not test subjects enrolled in the no‐TT group because of budgetary constraints. Subjects were seated with the trunk‐thigh angle at 90° and the knee flexed at 90° (where 0 corresponds to the full knee extension). Participants were securely strapped to the chair by two crossover shoulder harnesses and a belt across the hip joint. The axis of rotation of the dynamometer was aligned to the anatomical knee axis. The lever arm was attached 2–3 inches above the lateral malleolus. For isometric peak torque, surface NMES was applied to both knee extensor muscle groups after adjusting the current at 30 Hz and 450 µs. The current was set at 50 mA (two trials) and 100 mA (two trials) to test different muscle recruitment level. Each isometric trial was separated by 10–15 s of rest to avoid muscle fatigue with recurrent activation. Each participant was allowed 30–60 s of rest between either two consecutive isometric or isokinetic trials. After completion of isometric testing, participants were allowed approximately 5 min of rest before proceeding with isokinetic testing on the same leg. For isokinetic peak torques, knee extensor muscle group was tested at 60, 90, and 180 deg s^−1^ after setting the NMES current to 30 Hz, 450 µs, and 100–150 mA. Two trials were conducted per each speed and the measurements were conducted at DD0 and DD16 after closely matching the arc range of motion of knee flexion‐extension.

### 2.3 Statistical analyses

All data were tested for normality using the Shapiro–Wilk tests and if necessary (*p* < 0.05), data were then log‐transformed prior to any statistical analysis. Outliers were detected using normal Q–Q plots at different time points (DD0 and DD16) for each group. Mixed‐model ANOVA tests were performed to examine main time effects (DD0 and DD16) and between‐group (TT+RT vs. no‐TT) differences as well as interactions between groups on the primary (muscle CSAs) and secondary outcome variables over the course of the 16‐week DD period. If there was an interaction effect, post hoc analyses were then followed using independent *t*‐tests. To further dissociate a main time effect (DD0 and DD16), paired *t*‐tests were conducted within each group separately. Intent‐to‐treat analysis approach was adopted for those who experienced withdrawal during the trial by maintaining their group assignments. The approach was used to ensure the current study was not underpowered. Statistical analyses were performed using IBM‐SPSS version 26.0 (SPSS) and all values are presented as mean ± SD.

## 3 RESULTS

Physical and SCI characteristics were not different between the two groups during the 16‐week DD period (Table 1). Three participants from the TT+RT group (*n* = 9) withdrew from the training, because of conflict with either personal commitments or work. However, one out of the three participants agreed to participate in the DD0 and DD16 measurements without receiving testosterone patches or participating in the NMES‐RT (his data were included). The second and third participants withdrew after completion of week 1 and week 7, respectively (their data were not included in the analysis). Seven participants were considered for analysis in the TT+RT group. In the TT+RT group (*n* = 6), adherence to the progressive RT protocol was 96 ± 5% over 16 weeks.

**TABLE 1:** Physical and SCI characteristics of participants who enrolled in 16 weeks of dose de‐escalation period of either TT+RT or no‐TT. Values are presented as mean ± SD

Characteristics	TT+RT (*n* = 7)	no‐TT (*n* = 6)	*p*‐values (Time main effect/between‐groups effect/interaction effect)
Age (years)^a^	37 ± 11	35 ± 10	0.517
Weight (kg)‐DD0^a^	86.1 ± 18.6	76 ± 10.7	0.815/0.2247/0.297
Weight (kg)‐DD16	87.1 ± 20.4	74.5 ± 9.4	
Height (m)‐DD0^a^	1.80 ± 8	1.81 ± 5	0.630/0.995/0.220
Height (m)‐DD16	1.81 ± 8	1.80 ± 5	
BMI (kg/m^2^)‐DD0^a^	27.4 ± 4.2	23.2 ± 4.1	0.510/0.129/0.672
BMI (kg/m^2^)‐DD16	26.6 ± 5.8	23.0 ± 3.4	
Single neurological level (SNL)	C5‐T11 2 Tetraplegia 5 paraplegia	C6‐T6 4 Tetraplegia 2 paraplegia	–
TSI (years)	11 ± 11	9.5 ± 6.5	
ISNCSCI classification	A (*n* = 6) B (*n* = 1)	A (*n* = 4) B ( *n* = 2)	
Ethnicity	African American (*n* = 3) White (*n* = 4)	African American (*n* = 3) White (*n* = 3)	–

Abbreviations: BMI, body mass index; DD0, beginning of the 16 weeks of TT+RT or no‐TT interventions; DD16, end of dose de‐escalation of 16 weeks of TT+RT or no‐TT interventions; ISNCSCI, International Standards for Neurological Classification of Spinal Cord Injury; *n*, number; TSI, time since injury; TT, testosterone treatment group.

^a^ Independent *t*‐test to examine for statistical difference in age between both groups.

Across the 16 weeks, participants in the TT+RT managed to successfully complete the target number of sets (three sets) and repetitions (30 repetitions/per week) on both legs. Progression of the training for both lower extremities in the TT+RT group is listed in Table 2. Over the 16‐week period, weights were significantly decreased to 2 lbs. (Table 2). Q–Q plots detected outliers in transverse supine diameter, fasting insulin, and Si data and the data were excluded from further statistical analyses.

**TABLE 2:** Amplitude of the current required to evoke full knee extension and ankle weights (lbs.) lifted during 16 weeks of DD period in the TT+RT group

	Week 1‐DD	Week 4‐DD	Week 8‐DD	Week 12‐DD	Week 16‐DD
Right leg‐amplitude (mA)	131 ± 37	122 ± 36	101 ± 23	88.5 ± 27	99 ± 36
Left leg‐amplitude (mA)	120.5 ± 34.5	110 ± 37	97 ± 21.5	87 ± 28.5	94 ± 26.5
Right leg‐weights (Lbs.)	21 ± 6.5	15 ± 6.5	8.5 ± 4	3 ± 1	2
Left leg‐weights (Lbs.)	22 ± 3	16 ± 3	8.5 ± 3	3 ± 1	2

Fasting insulin, IL‐6, and CRP data at DD0 and DD16 did not meet the assumption of normality (i.e., Shapiro–Wilk test of *p* < 0.05) and were log‐transformed before conducting further statistical analyses.

### 3.1 Serum testosterone

Four days after removal of the patches, there was a significant main effect of time (*p* = 0.010) in both TT+RT (DD0: 171 ± 125, DD16: 304 ± 110 ng/dl) and no‐TT (DD0: 203 ± 160, DD16: 405 ± 160 ng/dl) groups, but not a significant group*time interaction (*p* = 0.25) for serum testosterone concentrations. Follow‐up paired *t*‐tests indicated that there was no difference in the TT+RT group (*p* = 0.100) and there was a trend (*p* = 0.064) within DD0 and DD16 in the no‐TT group.

#### 3.1.1 Body composition variables

##### Physical characteristics and anthropometrics

Physical characteristics (Table 1) and anthropometrics (Table 3) did not change in either TT+RT or no‐TT. There was a trend of increasing (*p* = 0.065) sagittal diameter in both groups (Table 3).

**TABLE 3:** Anthropometrics following 16 weeks of dose de‐escalation of TT+RT and no‐TT groups. Values are presented as mean ± SD

Characteristics	TT+RT (*n* = 7)	no‐TT (*n* = 6)	*p‐*values (time main effect/between‐groups effect/interaction effect)
Circumferences			
Sitting‐WC‐DD0 (cm)	93.6 ± 11.4	85.3 ± 5.7	0.487/0.144/0.849
Sitting‐WC‐DD16 (cm)	94.4 ± 12.3	85.7 ± 7.7	–
Supine‐WC‐DD0 (cm)	90.3 ± 14.6	81.2 ± 9.5	0.288/0.205/0.989
Supine‐WC‐DD16 (cm)	91.5 ± 14.3	82.4 ± 8.3	
Sitting‐AC‐DD0 (cm)	103.4 ± 17.6	97.1 ± 9.7	0.479/0.404/0.640
Sitting‐AC‐DD16 (cm)	105.0 ± 17	97.4 ± 11.3	
Supine‐AC‐DD0 (cm)	91.4 ± 16.7	84.8 ± 12.9	0.516/0.362/0.383
Supine‐AC‐DD16 (cm)	93.2 ± 16.2	84.5 ± 10.3	
Supine Hip‐DD0 (cm)	100.7 ± 14.2	95.8 ± 8.8	0.512/0.370/0.365
Supine Hip‐DD16 (cm)	102.7 ± 14.4	95.5 ± 6.0	
Sitting‐Calf Circ.‐DD0 (cm)	33.3 ± 4.8	32.7 ± 2.6	0.574/0.781/0.892
Sitting‐Calf Circ.‐DD16 (cm)	33.2 ± 5.3	32.5 ± 2.6	
Supine thigh Circ. DD0 (cm)	54.7 ± 9.8	48.5 ± 5.7	0.546/0.245/0.584
Supine thigh Circ. DD16 (cm)	54.1 ± 11.0	48.4 ± 6.8	
Diameters			
Sagittal sitting diameter‐DD0 (cm)	28.2 ± 5.1	27.5 ± 3.7	0.065/0.774/0.915
Sagittal sitting diameter‐DD16 (cm)	29.4 ± 5.4	28.6 ± 1.9	
Sagittal supine diameter‐DD0	23.9 ± 8.2	21.4 ± 4.0	0.981/0.483/0.558
Sagittal supine diameter‐DD16	23.1 ± 4.8	21.8 ± 3.2	
Transverse sitting diameter‐DD0 (cm)	33.9 ± 4.7	32.5 ± 2.7	0.289/0.485/0.670
Transverse sitting diameter‐DD16 (cm)	33.4 ± 4.4	31.5 ± 4.4	
Transverse supine diameter‐DD0 (cm)	30.6 ± 4.4	32.2 ± 4.6^a^	0.522/0.936/0.171
Transverse supine diameter‐DD16 (cm)	33.2 ± 4.7	31.2 ± 3.4	

Note

Supine position: measurements were taken while lying flat on the testing mat; sitting position; measurements were taken while sitting in their wheelchairs. Transverse diameter: measuring the largest length of the trunk in medio‐lateral direction; sagittal diameter: measuring the largest length of the trunk in antero‐posterior direction. Sagittal and transverse abdominal diameters were also measured at the level of the umbilicus using a Holtain–Kahn abdominal caliper.

Abbreviation: WC, waist circumference.

^a^ Outlier data of one participant were deleted from further statistical analyses (n=5) in the transverse supine diameter.

##### Body composition variables

Arm lean mass and FFM showed a significant time effect (*p* = 0.003) that indicated decline in both groups (Table 4). A between‐group effect was noted in leg lean mass (*p* = 0.018); a follow‐up independent *t*‐tests revealed differences in leg lean mass at DD0 (*p* = 0.005) and a trend at DD16 (*p* = 0.058).

**TABLE 4:** Body composition parameters as measured by DXA following 16 weeks of dose de‐escalation of TT+RT and TT only groups. Values are presented as mean ± SD

Characteristics	TT+RT (*n* = 7)	TT (*n* = 6)	*p*‐values (Time main effect /between‐groups effect/interaction effect)
Arms			
%FM‐DD0	24.2 ± 12.3	23.2 ± 7.7	0.503/0.727/0.343
%FM‐DD16	25.8 ± 11.0	22.8 ± 9.0	–
FM‐DD0 (kg)	2.58 ± 1.3	2.3 ± 0.97	0.591/0.557/0.454
FM‐DD16 (kg)	2.6 ± 0.98	2.2 ± 0.95	
Lean mass‐DD0 (kg)	7.6 ± 2.8	7.0 ± 1.9	0.003/0.657/0.880
Lean mass‐DD16 (kg)	7.3 ± 2.7	6.7 ± 1.8	
BMC‐DD0 (kg)	0.49 ± 0.12	0.46 ± 0.07	0.565/0.499/0.849
BMC‐DD16 (kg)	0.49 ± 0.09	0.45 ± 0.07	
FFM‐DD0 (kg)	8.1 ± 2.9	7.4 ± 2.0	0.003/0.649/0.880
FFM‐DD16 (kg)	7.8 ± 2.7	7.2 ± 1.8	
Legs			
%FM‐DD0	32.0 ± 9.5	39.2 ± 7.7	0.977/0.361/0.102
%FM‐DD16	34.4 ± 8.3	36.8 ± 11.3	
FM‐DD0 (kg)	8.9 ± 3.9	9.0 ± 2.8	0.512/0.856/0.231
FM‐DD16 (kg)	9.6 ± 4.4	8.8 ± 3.2	
Lean mass‐DD0 (kg)	17.0 ± 2.7	12.7 ± 1.2	0.259/0.018/0.283
Lean mass‐DD16 (kg)	16.15 ± 3.6	12.7 ± 1.7	
BMC‐DD0 (kg)	0.98 ± 0.20	0.86 ± 0.11	0.386/0.212/0.946
BMC‐DD16 (kg)	0.98 ± 4.8	0.85 ± 0.11	
FFM‐DD0 (kg)	18.0 ± 2.9	13.6 ± 1.3	0.260/0.020/0.290
FFM‐DD16 (kg)	17.1 ± 3.8	13.5 ± 1.7	
Trunk			
%FM‐DD0	36.5 ± 12.5	39 ± 10	0.764/0.867/0.223
%FM‐DD16	38.3 ± 9.7	38 ± 12	
FM‐DD0 (kg)	16.7 ± 8.5	15.3 ± 5.4	0.592/0.561/0.104
FM‐DD16 (kg)	17.9 ± 7.4	14.6 ± 6.0	
Lean mass‐DD0 (kg)	25.4 ± 4	22.0 ± 2.0	0.486/0.07/0.474
Lean mass‐DD16 (kg)	26.0 ± 4.4	22.0 ± 2.3	
BMC‐DD0 (kg)	1.13 ± 0.25	1.05 ± 0.28	0.650/0.527/0.809
BMC‐DD16 (kg)	1.12 ± 0.18	1.02 ± 0.31	
FFM‐DD0 (kg)	26.5 ± 4.2	23.0 ± 1.7	0.496/0.072/0.454
FFM‐DD16 (kg)	27.1 ± 4.5	23.0 ± 2.3	
Total			
%FM‐DD0	32.3 ± 11	35.6 ± 8.5	0.513/0.735/0.130
%FM‐DD16	34.4 ± 8.8	34.7 ± 10.2	
FM‐DD0 (kg)	29.2 ± 14.0	27.6 ± 9.4	0.575/0.645/0.092
FM‐DD16 (kg)	31.15 ± 12.6	26.6 ± 10.3	
Lean mass‐DD0 (kg)	54.2 ± 8.7	45.4 ± 4.0	0.501/0.054/0.746
Lean mass‐DD16 (kg)	53.5 ± 9.6	45.2 ± 3.6	
BMC‐DD0 (kg)	3.2 ± 0.52	2.9 ± 0.48	0.511/0.342/0.936
BMC‐DD16 (kg)	3.2 ± 0.44	2.9 ± 0.51	
FFM‐DD0 (kg)	57.4 ± 9.1	48.3 ± 4.2	0.466/0.058/0.735
FFM‐DD16 (kg)	56.7 ± 10.1	48.1 ± 3.7	

Abbreviations: %FM: percentage fat mass; BMC, bone mineral content; DD0, measurements before initiating a dose de‐escalation period; DD16, measurements after 16 weeks of dose de‐escalation period; FFM, fat‐free mass; FM, fat mass.

There was a trend of interaction in total body FM between TT+RT and no‐TT groups (*p* = 0.09).

##### Skeletal muscle and IMF CSAs

###### Thigh muscle CSA

Table 5 demonstrates the changes in thigh skeletal muscle CSAs following TT+RT and no‐TT following the 16‐week DD phase.

**TABLE 5:** Whole thigh CSA, whole and absolute muscle CSA, and intramuscular fat in persons with SCI for participants following 16 weeks of dose de‐escalation of TT+RT and no‐TT groups

Anatomical region	TRT+RT (*n* = 7)				no‐TT (*n* = 6)			
	Right‐DD0	Right‐DD16	Left‐DD0	Left‐DD16	Right‐DD0	Right‐DD16	Left‐DD0	Left‐DD16
Whole thigh CSA (cm^2^)								
A1 (#1–4)	259 ± 77	256 ± 94	247 ± 80	244 ± 80	202 ± 45	204 ± 52	202 ± 52	202 ± 60
A2 (#5‐8)	217 ± 75	215 ± 81	212 ± 71	208 ± 73	165 ± 38	169 ± 44	169 ± 45	169 ± 50
A3 (#9–12)	171 ± 59	170.4 ± 63	169.3 ± 59	168 ± 61	137 ± 35	138 ± 36	138.5 ± 39	136 ± 38
Average	214.5 ± 72	213 ± 79	208 ± 69	206 ± 71	167 ± 37.5	169 ± 42.3	168 ± 43.5	167 ± 48
Whole thigh muscle CSA‐SAT (cm^2^)								
A1 (#1–4)	140 ± 36	132 ± 39*^X^	135 ± 30	127 ± 30*^#′^	101 ± 21.4	106 ± 26	97 ± 24	101 ± 28
A2 (#5–8)	131 ± 32	124 ± 35.5^X^	130 ± 33	124 ± 35*^#′^	92.5 ± 21	97.5 ± 25.5	90 ± 23.5	94 ± 28
A3 (#9‐12)	107 ± 25	105 ± 29	108.5 ± 30	105 ± 34^#′^	78 ± 19	82 ± 21	77 ± 20	80 ± 22
Average	125 ± 31	120 ± 35*^X^	124 ± 31.5	119 ± 33*^#′^	90 ± 19	94.5 ± 23	87.5 ± 22	91 ± 25
Absolute whole thigh muscle CSA‐IMF (cm^2^)								
A1 (#1–4)	126.3 ± 31	116 ± 32.5*^x^	122 ± 30	114 ± 27.7*^#′^	91 ± 17	95 ± 21	87 ± 18.5	89.2 ± 23.2
A2 (#5–8)	116 ± 25	108 ± 27*^x^	115.4 ± 29	110 ± 29*^#^	81 ± 18	84 ± 22	78.6 ± 19.0	80.4 ± 22.7
A3 (#9–12)	91 ± 18.1	88 ± 21.4	92.4 ± 24.3	90.4 ± 28*^#^	66 ± 17.5	65.3 ± 18.5	65.0 ± 17.4	65 ± 20
Average	110.7 ± 25	104 ± 27*^x′^	110 ± 28	105 ± 27.5*^#^	78.3 ± 15.5	81.2 ± 19	76.2 ± 17	77.6 ± 21
Whole knee extensor CSA (cm^2^)								
A1 (#1–4)	66.5 ± 15	60 ± 17.5*′^#^	63.4 ± 11.4	58 ± 15.5*^′#^	47 ± 17	42.5 ± 8.5*′	44 ± 17	40 ± 10.4*′
A2 (#5–8)	64.6 ± 12	60 ± 17.5*′^#^	63.8 ± 14	59.2 ± 15.7*^′#^	45.5 ± 16.2	42 ± 8.2*′	44.3 ± 16.4	40.5 ± 10.0*′
A3 (#9–12)	52.2 ± 7.8	49.8 ± 11.4^#^	52 ± 12	49.3 ± 14.7^#^	37.2 ± 12.8	34.4 ± 7.4	36.3 ± 12.7	34.3 ± 8.4
Average	61 ± 12	56 ± 14*′^#^	59.5 ± 14.7	55.6 ± 15*^′#^	43.0 ± 15.0	39.3 ± 7.6*′	41.4 ± 15.3	38.0 ± 9.2*′
Absolute knee extensor CSA‐IMF (cm^2^)								
A1 (#1–4)	63.6 ± 15	56.7 ± 16.5*^x#^	61 ± 5.5	56 ± 15 ^x#^	39.3 ± 10.6	40.7 ± 8.0	37.0 ± 7.2	38.0 ± 9.3
A2 (#5–8)	60.5 ± 11.5	56 ± 13.4*^x#^	61 ± 5.5	56 ± 15 ^x#^	37.2 ± 5.7	38.7 ± 8.0	36.3 ± 7.5	37.5 ± 8.9
A3 (#9–12)	47.8 ± 7.6	46 ± 10.5*^x#^	47.6 ± 10	45 ± 14 ^x#^	30.4 ± 7.4	31.4 ± 7.2	30.2 ± 8.0	31.8 ± 7.9
Average	57 ± 11	53 ± 13*^x#^	56 ± 13.5	52.5 ± 14 ^x#^	35.3 ± 5.3	36.6 ± 7.5	34.3 ± 7.0	35.6 ± 8.4
Adductor m. CSA								
A1 (#1–4)	36 ± 9.0	33.3 ± 8.8*	35.2 ± 6.2	32.3 ± 5*	28.4 ± 8.6	29.9 ± 10.6	28.0 ± 9.0	29.1 ± 10.7
A2 (#5–8)	16.7 ± 4.0	15.7 ± 3.8	19.0 ± 5.3	16.7 ± 5.5	12.6 ± 7.3	14.7 ± 9.5	13.2 ± 7.4	14.6 ± 8.7
A3 (#9–12)	2.8 ± 2.0	2.4 ± 1.4	4.1 ± 2.3	3.2 ± 2.5	2.3 ± 3.2	3.2 ± 4.1	3.2 ± 3.5	3.4 ± 4.3
Average	18.1 ± 4.3	17.1 ± 4.6	19.2 ± 4.4	17.4 ± 3.6	13.8 ± 5.1	15.15 ± 6.7	14.1 ± 5.4	15.1 ± 6.4
Knee flexor m. CSA								
A1 (#1–4)	12.4 ± 4.2	12.2 ± 4.3	13.4 ± 5.2	12.8 ± 4.7	12.3 ± 3.4	11.6 ± 2.1	11.4 ± 2.9	10.9 ± 2.5
A2 (#5–8)	26.7 ± 10.3	26.0 ± 11.0	25.6 ± 10.0	25.1 ± 8.4	21.7 ± 5.8	21.6 ± 6.1	20.2 ± 5.2	20.0 ± 6.7
A3 (#9–12)	30.2 ± 9.0	30.2 ± 9.7	30.5 ± 9.4	30.4 ± 10.2	24.4 ± 6.8	23.7 ± 7.6	22.8 ± 4.9	23.3 ± 6.4
Average	23.2 ± 7.7	22.8 ± 8.3	23.6 ± 8.0	22.7 ± 7.8	19.5 ± 5.3	19.6 ± 5.3	18.3 ± 4.2	18.3 ± 5.0

Note

Abbreviations: CSA, cross‐sectional area; IMF, intramuscular fat; SAT, subcutaneous adipose tissue.

*, Statistical difference from DD0, whole thigh muscle CSA (*p* < 0.0001); x, statistical interaction between TT+RT and no‐TT groups for whole thigh muscle CSA (*p* < 0.0001); x′, trends toward interaction; #, between‐group differences (*p* < 0.05); #′, trend between both groups (*p* = 0.07–0.09).

A1: area 1 (#1–4), reflects the anatomical (A) region of the average of the proximal four CSA slices of the thigh immediately following the inferior border of the gluteus maximus muscle; A2: area 2 (#5–8), reflects the average CSA of the mid four MRI slices of the thigh; A3: area 3 (#9–12), reflects the average CSA of the distal four MRI slices of thigh toward the knee joint; Average: reflects the average CSA of 12 MRI slices of the entire thigh.

###### Absolute thigh muscle CSA

Absolute thigh muscle CSAs (i.e., after excluding IMF) showed interaction effects in the proximal right slices (TT+RT: −8.15% and no‐TT:4.4%; *p* = 0.02), mid right slices (TT+RT: −6.9% and no‐TT:3.7%; *p* = 0.031), and proximal left slices (TT+RT: −6.5% and no‐TT:2.3%; *p* = 0.049). There was also a trend (*p* = 0.056) of an interaction in the average absolute thigh muscle CSA between TT+RT and no‐TT groups (Table 5). Additionally, there were between‐group effects in proximal four slices (*p* = 0.081–0.057), mid four slices (*p* = 0.04–0.037), distal four slices (*p* = 0.038–0.058), and the average (*p* = 0.048–0.0043) absolute thigh muscle CSA between TT+RT and no‐TT groups. Follow‐up independent *t*‐tests revealed that the proximal slices (*p* = 0.03), mid slices (*p* = 0.05–0.23), distal (*p* = 0.039 −0.050), and average (*p* = 0.034–0.035) absolute thigh absolute muscle CSA were significantly different between TT+RT and no‐TT groups at DD0 but not at DD16.

#### 3.1.2 Individual muscle CSA

##### Absolute knee extensor muscle CSA

There was an interaction effect between groups in the proximal right (TT+RT: −11% and no‐TT: 3.5%; *p* = 0.024) and proximal left (TT+RT: −8% and no‐TT:2.6%; *p* = 0.01), right‐mid (TT+RT:‐7.3% and no‐TT: 2.6%; *p* = 0.053) and left‐mid (TT+RT: −7.0% and no‐TT: 2.7%; *p* = 0.07), and right average (TT+RT: −7.1% and no‐TT;3.7% *p* = 0.051) and left (TT+RT: −6.3% and no‐TT: 3.8%; *p* = 0.055) absolute knee extensor muscle CSA. There were also between‐group effects in the proximal slices (right: *p* = 0.012 and left: *p* = 0.012), mid slices (right: *p* = 0.004 and left: *p* = 0.012), distal slices (right: *p* = 0.004 and left: *p* = 0.008), and average (right: *p* = 0.006 and left: *p* = 0.01) absolute knee extensor CSAs (Table 5).

##### Knee flexors and hip adductor muscle CSA

There was a trend of an interaction between TT+RT and no‐TT groups in left adductor muscle CSA (9.9% at DD16, *p* = 0.053; Table 5), without changes in the right adductor muscle CSA. Knee flexor muscle CSAs did not show any changes within or between groups (*p* > 0.05).

##### Visceral and subcutaneous adiposity

**FIGURE 3:**
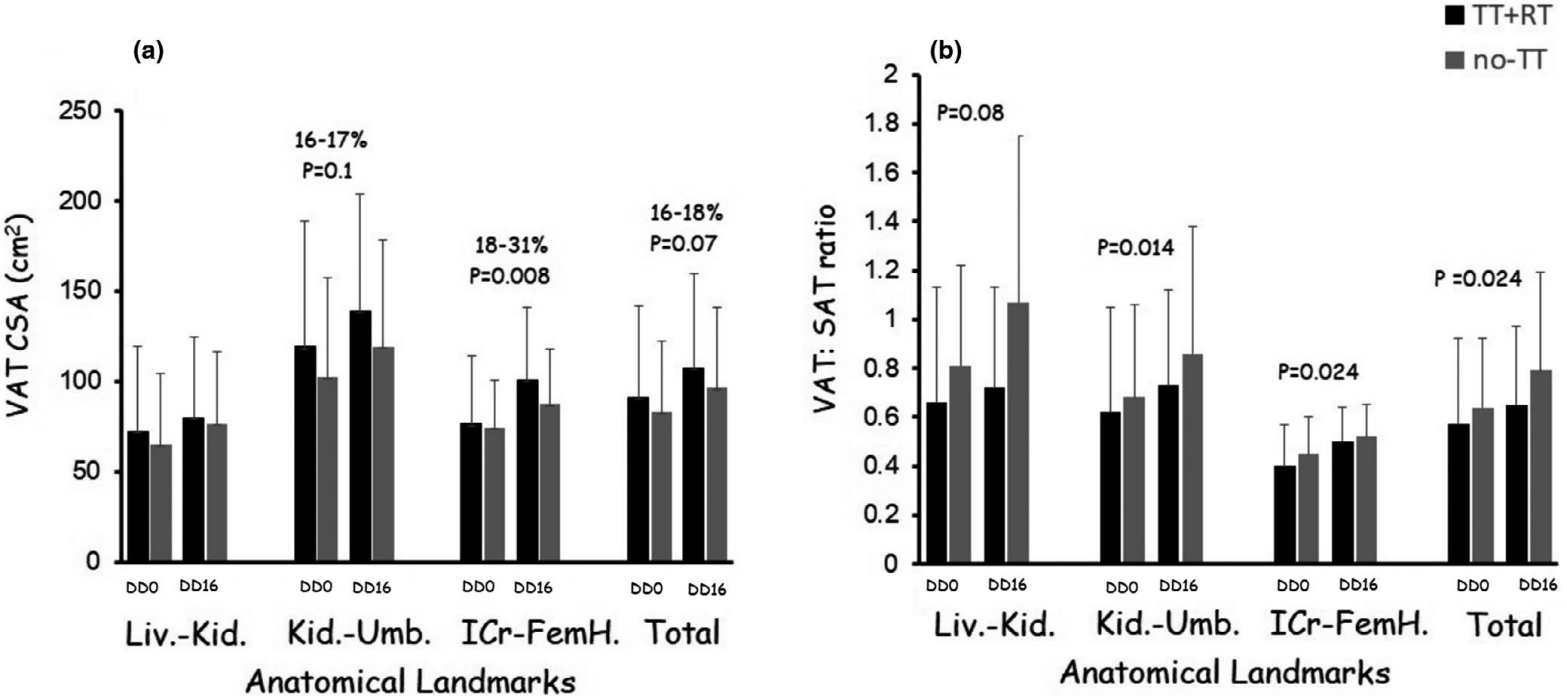
Changes in (a) VAT CSA and (b) VAT:SAT ratio following 16 weeks of DD period in the TT+RT and no‐TT groups. Considering the non‐uniform distribution of VAT, VAT CSAs were measured at different anatomical landmarks including the regions between liver and kidneys (Liv.‐Kid.), kidneys and umbilicus (Kid.‐Umb.), iliac crests and femoral heads (IC‐FH), and finally the average of all trunk MRI slices (total VAT). P‐values reflected main effects of time. CSA, cross‐sectional area; DD, dose de‐escalation; MRI, magnetic resonance imaging; RT, resistance training; SAT, subcutaneous adipose tissue; TT, testosterone treatment; VAT, visceral adipose tissue

Figure 3a,b presents VAT CSA and VAT:SAT ratio across different anatomical trunk landmarks in persons with SCI. There were no interactions for VAT CSA or VAT: SAT ratio (*p* > 0.05). There were no differences between TT+RT and no‐TT groups. SAT_L‐k, k‐U, IC‐FH_ remained unchanged (*p* > 0.05) in both the TT+RT (184±121 to 191±107 cm^2^) and no‐TT (155 ± 67 to 149 ± 66.5 cm^2^) groups.

#### 3.1.3 Metabolic profile

##### Blood pressure and heart rate

There were no main effect of time or between‐group differences in resting systolic (TT+RT: 114±18 to 115±22 mmHg and no‐TT: 100 ± 8 to 101 ± 14 mmHg; *p*s = 0.845 or 0.109, respectively), resting diastolic (TT+RT: 73 ± 13 to 72 ± 11 mmHg and no‐TT: 61 ± 9 to 63 ± 10 mmHg; *p*s = 0.837 or 0.058, respectively), and resting heart rate (TT+RT: 77 ± 21 to 84 ± 22 bpm and no‐TT: 74 ± 13 to 78 ± 24 bpm; *p*s = 0.120 or 0.672, respectively) during the 16‐week DD period or any interaction between groups (*p* > 0.05).

##### Basal metabolic rate

There was no main time effect (*p* = 0.140) or between‐groups effect (*p* = 0.178) in BMR. Contrary, there was an interaction (*p* = 0.005) between TT+RT (−3.7%: 1646 ± 196 to 1584 ± 223 kcal day^−1^) and no‐TT (9.0%: 1310 ± 223 to 1431 ± 299 kcal day^−1^) groups in BMR. A follow‐up independent *t*‐tests revealed that there was a difference between TT+RT and no‐TT groups at DD0 (*p* = 0.015) but not at DD16.

##### Carbohydrate, lipid, and inflammatory biomarker metabolism

Carbohydrate, lipid, and inflammatory biomarker profiles are presented in Table S1. Neither groups (TT+RT vs. no‐TT) showed any changes (*p* > 0.05) in fasting insulin, fasting glucose, insulin sensitivity (Si), glucose effectiveness (Sg), HBA_1c_ (%), lipid panel, CRP, TNF‐α, IL‐6, or free fatty acids.

#### 3.1.4 Peak isometric and isokinetic torques

In the TT+RT group (*n* = 7), there were no changes (*p* > 0.05) in peak isometric torques at 50 mA or 100 mA following 16 weeks of the DD period. The slowness in the rise time was also maintained at both 50 mA (0.12 ± 0.016 to 0.11 ± 0.02 ms, *p* = 0.1) and 100 mA (0.13 ± 0.007 to 0.15 ± 0.013 ms, *p* = 0.2). Isokinetic peak torques were also maintained (*p* > 0.05) at 60, 90, and 180 deg s^−1^ in the TT+RT group.

## 4 DISCUSSION

The major findings of the current study indicated that 16 weeks of DD program of adding low‐dose TT to NMES‐RT was minimally effective in maintaining the effects of determining on muscle size and BMR compared to the no‐TT group. Participants in both groups experienced increases in VAT without noticeable changes in the cardiovascular, carbohydrate, lipid, or inflammatory biomarker profiles. Cessation of administering testosterone resulted in a continuous decrease in muscle CSA and increase in the VAT CSA in the no‐TT group. Furthermore, the addition of 2 mg day^−1^ TT to NMES‐RT did not maintain the increase in muscle size or the decrease in VAT that was previously observed following 16 weeks of TT+RT compared to TT only (Gorgey, Khalil, et al., 2019). Both groups did not show additional changes in body composition or metabolic profile over the 16 weeks of detraining. Finally, the de‐escalation training was successful in retaining the neuromuscular adaptions previously demonstrated by increasing peak isometric and isokinetic torques as well as slowness in the rise time following TT+RT.

The current study adopted a novel strategy of detraining via administering a low‐dose TT (2 mg day^−1^) and reciprocally regressed the weightlifting program by decreasing the frequency of NMES‐RT from twice to once weekly and decreasing ankle weights by 2 lbs. per week. At the end of week 12, participants in the TT+RT performed 30 reps per leg and decreased weights to 2 lbs. and continued training for additional 4 weeks with the same weights. Similar to previous work (Bickel et al., 2011), we aimed to decrease the training volume by reducing the number of sets and the frequency of the training. In the first phase of the study, participants were able to lift on average 20 lbs. per leg over a 16‐week period (Gorgey, Khalil, et al., 2019). Considering that the person with SCI is a model of aging (O'Brien, Wade, et al., 2017), it is interesting to note the similarity between the current findings and previous work in able‐bodied subjects (Bickel et al., 2011). Previous work noted that a DD of 1/3 or 1/9 of the initial RT volume did not successfully maintain the gains in myofiber size or type in older adults; however, muscle strength was preserved (Bickel et al., 2011). Furthermore, both groups experienced rebound effects on their circulating endogenous testosterone after significantly reduced the dose of transdermal patches from 2–6 mg day^−1^ during the initial training phase (Gorgey, Khalil, et al., 2019). During the DD phase, the low‐dose of 2 mg day^−1^ was not sufficient to inhibit endogenous testosterone in the TT+RT group. This rebound effect in endogenous testosterone may explain the modest non‐significant increases in muscle CSA and BMR in the no‐TT group.

In the current work, TT+RT was minimally effective in preventing the loss in muscle mass. In the first phase, participants gained approximately more than 30%–40% in absolute muscle CSAs and they lost close to 10% in the second detraining phase. Regressing the frequency, repetitions, and ankle weights over the 16‐week period of DD eventually resulted in decreasing the volume of the training necessary to evoke protein accretion and muscle hypertrophy. Others have shown that the gains in muscle mass and trabecular bone parameters were partially preserved following cessation of high‐volume functional electrical stimulation cycling for 1 year in persons with SCI (Frotzler et al., 2009). Another recent study showed that 3 weeks of detraining resulted in decreasing muscle thickness to initial baseline following 8 weeks of combined functional electrical stimulation exercise with blood flow restriction in persons with complete SCI (Skiba et al., 2021).

We have previously shown in the same group the activation of phosphorylated AKT as a molecular signal of muscle hypertrophy (Gorgey et al., 2020). Decreasing the volume of NMES‐RT may abruptly deactivate the signaling pathways necessary to maintain the previously gained muscle hypertrophy. On the other hand, the no‐TT group did not receive any TT over the 16‐week DD period similar to previous work (Bauman et al., 2015). Bauman et al. (2015) showed that despite cessation of TT following 12 months, hypogonadal participants with SCI maintained the gain in lean mass and resting metabolic rate for additional 6 months. In the initial 16 weeks of this trial, participants enrolled in the TT only group showed a modest decrease in VAT (Gorgey, Khalil, et al., 2019). Unlike the findings of Bauman et al., cessation of TT resulted in reciprocal increase in VAT in both groups.

Magnetic resonance imaging revealed robust changes in muscle size and VAT during the 16‐week detraining period. Furthermore, MRI facilitated the measure of whole muscle CSA and absolute muscle CSA after subtracting IMF. We intentionally did not average data in both the right and left thigh muscles to ensure that the effect of detraining was even and because each leg was trained separately (Mahoney et al., 2005); especially with the fact that the magnitude of ankle weights was slightly different at the end of initial phase of the trial (Gorgey, Khalil, et al., 2019). There were no differences as result of the detraining between the right and left legs. DXA did not demonstrate major changes in body composition over the 16‐week DD period. However, DXA has previously been used to study the changes in legs and total body lean mass after more than 2 years of detraining in persons with complete SCI (Gorgey, Martin, et al., 2016). This may explain previous research findings that showed retention of gains in lean mass following 6 months of cessation of testosterone after using variable dose for 12 months in hypogonadal men with SCI (Bauman et al., 2015). The discrepancy between the studies could be simply explained using different imaging techniques. Another important determination is that 73% had low serum testosterone levels in the TT‐RT group (215–313 ng/dl) and 64% had low testosterone levels in the no‐TT group (140–402 ng/dl; Gorgey, Khalil, et al., 2019). Differences in gonadal status between the studies may have required a higher dose of testosterone (5–10 mg day^−1^) with longer carryover effects in men with SCI (Bauman et al., 2015).

The current findings suggest that a DD program of TT+RT and cessation of TT were not successful in retaining the decrease in VAT in persons with SCI. The increase in VAT could be simply explained by decreasing level of physical activity after SCI. VAT imposes significant cardiometabolic risks in persons with SCI (Farkas et al., 2017; Gill et al., 2020). Several studies suggested that VAT is associated with a spectrum of inflammatory biomarkers and likely impacts hepatic metabolism and causes mitochondrial dysfunction (Farkas et al., 2017; O'Brien, Wade, et al., 2017; Sumrell et al., 2018). The increase in the level of physical activity and NMES‐RT successfully reduce VAT in persons with SCI (Gorgey, Khalil, et al., 2019; Pelletier et al., 2018). During the initial 16‐week training period in these participants, both TT+RT and TT only modestly decreased VAT CSA. In a previous case report, the authors demonstrated that dietary manipulation via caloric restriction for 8 weeks combined with TT remarkably decreased both trunk VAT and SAT (Gorgey et al., 2019).

The effects of detraining on metabolic profile were recognized in a 3.9% decline in BMR in the TT+RT group. The initial phase showed a 17% increase in BMR following 16 weeks of TT+RT (Gorgey, Khalil, et al., 2019). It is important to note that BMR was non‐significantly higher at DD16 in the no‐TT group; this could be simply attributed to increase in endogenous testosterone following cessation of administration of TT in this group (Welle et al., 1992). In the TT+RT group, the loss in muscle mass was accompanied with a non‐significant decrease in BMR. Previously, it was noted that adiponectin, an insulin‐sensitizing hormone, may drive the increase in BMR via an increase in mitochondrial citrate synthase (O'Brien et al., 2018). Persons with SCI have greater levels of circulating adiponectin compared to able‐bodied controls (Maruyama et al., 2008; Wang et al., 2005). We have further demonstrated that addition of TT to NMES‐RT resulted in decreasing circulating adiponectin (Gorgey, Khalil, et al., 2019). This higher level of adiponectin may compensate for the loss in sympathetic nervous system and drive an increase in BMR in persons with SCI (O'Brien et al., 2018); which may explain the higher levels in persons with SCI. NMES‐RT‐induced skeletal muscle hypertrophy and increases BMR. The increase in BMR may result in a feed‐forward mechanism that suppresses adiponectin levels in persons with SCI (Gorgey, Khalil, et al., 2019).

The gain in muscle size following 16 weeks of training using TT+RT was mirrored with increases in peak torque, specific tension of the trained knee extensor muscles, and slowness of the rise time (Holman & Gorgey, 2019). The increase in specific tension reflected increases in both neural drive and muscle hypertrophy of the knee extensors. Previous reports indicated that detraining is associated with reductions in maximal voluntary contraction (Colliander & Tesch, 1992; Narici et al., 1989), muscle CSA (Narici et al., 1989), and neural drive to the muscle (Narici et al., 1989). In the current study, NMES‐RT combined with low dose of TT was successful in maintaining the gains in peak isomeric and isokinetic torques as well as the slowness of the rise time that were previously noted in the initial phase of the trial. The findings are in line with previous work that suggested that neuromuscular adaptions were preserved during months of detraining following heavy RT (Andersen et al., 2005).

### 4.1 Limitations and future directions

In addition to the limitations of our previous work (Gorgey, Khalil, et al., 2019), the current study had a small sample size. Due to budgetary constraints, it was extremely difficult to retain participants across the 16‐week training and 16‐week detraining period of the study. We only managed to enroll more than 50% of the sample size that were enrolled in the initial 16‐week training phase (Gorgey, Khalil, et al., 2019). This also limited our ability to measure neural adaptions in the no‐TT group. Additionally, three of the participants in the TT+RT group withdrew from participating in the 16‐week period of DD. However, one participant agreed to be enrolled in the DD0 and DD16 measurements. We have included his data with the other six participants because the primary research question was aimed toward understanding the effects of detraining on cardiometabolic profiles and neuromuscular parameters in persons with SCI. In the current work, we have defined detraining as either decrease in the volume or dose of the intervention (TT+RT group) or complete cessation of the designed intervention (no‐TT group). Additionally, carefully screening his data did not indicate noticeable changes to be considered as an outlier compared to the mean of the other six participants in the TT+RT group.

Another concern is the failure to account for their dietary habits during the training period. It is possible that the participants adapted poor dietary habits that offset the carryover effects of TT+RT or TT in men with SCI.

The current findings highlighted the need to develop rehabilitation or pharmaceutical approaches to attenuate the loss in lean mass during a detraining program. Alternating leg exercise with an arm cycling exercise or circuit RT programs may encourage long‐term compliance and allow participants to increase their daily level of physical activity. Leisure time physical activity has been shown to attenuate the development of chronic comorbidities and the gain in VAT in persons with SCI (Buchholz et al., 2009; Pelletier et al., 2018).

## 5 CONCLUSIONS

A follow‐up DD training program of TT+RT or no‐TT was minimally effective in preventing loss in muscle size, decreasing BMR, and increasing VAT. These findings expand our understanding of how muscle size, BMR, and VAT are changed during a DD of testosterone and NMES‐RT doses in persons with SCI. The findings further suggest that neuromuscular adaptations were retained in men with SCI; highlighting possible discordance between spinal cord circuity and muscle adaptations following detraining in men with SCI. Considering the prevalence of cardiometabolic disorders in this population, the current findings are helpful for designing future studies to explore the effects of longitudinal trials via applications of telehealth technology to ensure adherence and long‐term compliance.

## CONFLICT OF INTEREST

The authors have no competing interests to declare.

## AUTHORS' CONTRIBUTION

Ashraf S. Gorgey: Conceived the idea, secured funding, data collection, data analysis, and drafting manuscript; Refka E. Khalil: Subjects; recruitment, data collection, and data management; Ranjodh Gill: Administering metabolic test (IVGTT), data analysis, and drafting manuscript; M. Rehan Khan: Facilitate MRI capturing and oversee completion of the radiological scans, data collection and management; Robert A Adler: Medical monitor person, prescription of testosterone, oversee subjects' across the trial, data analysis, and drafting manuscript.

## Supporting information



Table S1
