# Supplementary material for: Effects of dose de‐escalation following testosterone treatment and evoked resistance exercise on body composition, metabolic profile, and neuromuscular parameters in persons with spinal cord injury
Source: Physiol Rep. 2021 Oct 29;9(21):e15089. doi: 10.14814/phy2.15089 (PMC8554770; doi:10.14814/phy2.15089)
Supplement: Supplementary file 1 — Table S1 [file PHY2-9-e15089-s001.docx]

**Table 1supplement**. Lipid, carbohydrate and inflammatory biomarker profiles following 16 weeks of dose de-escalation of TT+RT and no-TT groups. Values are presented as mean ± standard deviation (SD).

|  | **Characteristics** | **TT+RT**  ***(n=7)*** | **no-TT**  ***(n=6)*** | ***P-values***  *(Time main effect /between groups effect/interaction effect)* |
| --- | --- | --- | --- | --- |
| ***Lipid Profile*** | ***LDL-DD0***  (mg/dl) | 113.4±36.0 | 94.5±30.1 | 0.495/ 0.298/0.976 |
|  | ***LDL-16***  (mg/dl) | 109.4±34.8 | 90.8±26.4 | - |
|  | ***HDL-DD0***  (mg/dl) | 37.1±8.3 | 36.0±7.8 | 0.329/0.908/0.570 |
|  | ***HDL-DD16***  (mg/dl) | 39.2±8.9 | 35.0±10.5 |  |
|  | ***Chol-DD0***  (mg/dl) | 178.0±40.6 | 151.5±26.0 | 0.266/0.193/0.929 |
|  | ***Chol-DD16***  (mg/dl) | 168.8±41.7 | 143.6±30.8 |  |
|  | ***Chol: HDL Ratio DD0*** | 4.85±0.81 | 4.74±0.99 | 0.709/0.232/0.883 |
|  | ***Chol: HDL Ratio DD16*** | 4.20±1.27 | 4.15±0.61 |  |
|  | ***TG-DD0***  (mg/dl) | 136.85±62.5 | 94.66±19.70 | 0.303/0.615/0.615 |
|  | ***TG-DD16***  (mg/dl) | 117.42±40.2 | 87.83±26.10 |  |
| ***Carbohydrate Profile*** | ***Fasting Glucose DD0***  (mg/dl) | 94.6±11.3 | 86.0±6.6 | 0.307/0.517/0.323 |
|  | ***Fasting Glucose DD16***  (mg/dl) | 82.58±19.5 | 85.8±6.4 |  |
|  | ***Fasting Insulin DD0***  (µU/ml) | 4.48±2.89 | 2.47±1.09 | 0.893/0.488/0.096 |
|  | ***Fasting Insulin DD16***  (µU/ml) | 3.06±1.12 | 3.68±2.54 |  |
|  | ***SgDD0 (n=6)^***  *min^-1^* | 0.0178±0.0104 | 0.0256±0.018 | 0.888/0.194/0.747 |
|  | ***SgDD16 (n=4)^***  *min^-1^* | 0.0171±0.0099 | 0.0282±0.0177 |  |
|  | ***SiDD0 (n=6)^***  **(**min^-1^(µU/ml)^-1^) | 4.56±2.26 | 4.34±1.21 | 0.527/0.591/0.386 |
|  | ***SiDD16 (n=4)^***  **(**min^-1^(µU/ml)^-1^) | 4.34±1.211 | 6.15±297 |  |
|  | ***HbA1C (%)-DD0*** | 5.28±0.37 | 5.31±0.42 | 0.161/0.810/0.072 |
|  | ***HbA1C (%)-DD16*** | 5.43±0.20 | 5.25±0.15 |  |
| ***Inflammatory Biomarkers*** | ***CRP-C DD0***  (ng/ml) | 7526.2±7270.4 | 5316.6±3403.7 | 0.705/0.705/0.570 |
|  | ***CRP-C DD16***  (ng/ml) | 6019.4±6640.5 | 5629.0±6576.4 |  |
|  | ***IL-6 DD0***  (pg/ml) | 3.194±6.4 | 4.74±5.25 | 0.481/0.321/0.366 |
|  | ***IL-6 DD16***  (pg/ml) | 2.748±2.92 | 8.22±9.14 |  |
|  | ***TNF-α DD0***  (pg/ml) | 14.6±8.92 | 12.8±9.95 | 0.395/0.665/0.937 |
|  | ***TNF-α DD16***  (pg/ml) | 16.92±3.49 | 14.70±9.08 |  |
|  | ***FFA C-DD0***  (mmol/L) | 405.6±189.5 | 397.5±138.9 | \| 0.599/0.927/0.791 \| \| --- \| |
|  | ***FFA C-DD16***  (mmol/L) | 82.58±105.4 | 397.5±138.9 |  |

***LDL****: low density lipoprotein;* ***HDL****: high density lipoprotein;* ***Chol****: cholesterol;*

***TG****: triglycerides;* ***Sg****: glucose effectiveness;* ***Si****:insulin sensitivity;* ***HbA1c (%)****: percentage haemoglobin A1c;* ***CRP****: c-reactive protein;* ***IL6****: interlukin 6;* ***TNF-α****: tumor necrosis factor alpha; ^, outliers were detected and ommitted from further statistical analyses.*
